# A co-culture nanofibre scaffold model of neural cell degeneration in relevance to Parkinson’s disease

**DOI:** 10.1038/s41598-020-59310-x

**Published:** 2020-02-17

**Authors:** Joseph M. Chemmarappally, Henry C. N. Pegram, Neranga Abeywickrama, Enzo Fornari, Alan J. Hargreaves, Luigi A. De Girolamo, Bob Stevens

**Affiliations:** 1 Innovations in Surfaces, Materials and Related Technologies (iSMART), College of Science and Technology, Nottingham Trent University, Clifton, NG11 8NS UK; 2 Interdisciplinary Biomedical Research Centre (IBRC), College of Science and Technology, Nottingham Trent University, Clifton, NG11 8NS UK

**Keywords:** Neurological models, Parkinson's disease

## Abstract

Current therapeutic strategies for Parkinson’s disease (PD) aim to delay progression or replace damaged neurons by restoring the original neuronal structures. The poor regenerative capacity of neural tissue highlights the need for the development of cellular environments to model the pathogenesis of PD. In the current work, we have characterised the growth, survival and response to PD mimetics of human SH-SY5Y neuroblastoma and U-87MG glioblastoma cell lines cultured on polyacrylonitrile (PAN) and Jeffamine® doped polyacrylonitrile (PJ) nano-scaffolds. Differentiation induced by a range of agents was evaluated by immunoassays of neural protein biomarkers. PAN and PJ nanofibre scaffolds provided suitable three-dimensional (3D) environment to support the growth, differentiation and network formation of dopaminergic neuron- and astrocyte-like cell populations, respectively. The scaffolds selectively supported the survival and differentiation of both cell populations with prolonged neuronal survival when exposed to PD mimetics in the presence of astrocytes in a co-culture model. Such 3D nanoscaffold-based assays could aid our understanding of the molecular basis of PD mimetic-induced Parkinsonism and the discovery of neuroprotective agents.

## Introduction

Parkinson’s disease (PD), a progressive neurodegenerative disorder, is caused by loss of dopaminergic neurons in the *substantia nigra* of the midbrain and affects 1–2% of the population over 65 years of age^1^. Cells of the *substantia nigra* produce the neurotransmitter dopamine to control and coordinate motor functions. Their loss results in Parkinsonism, which manifests itself as muscle rigidity, tremors, slowness and difficulty in controlling movement^2^. Despite the prevalence of PD and the substantial efforts in studying disease pathogenesis, very limited disease-modifying agents exist. Current strategies only delay disease progression while novel proposed approaches attempt to reverse dopaminergic neuronal loss by implantation of human embryonic stem cells to restore neuronal architecture and promote neurite regeneration^3,4^. The development of new treatments is hampered by the scarcity of suitable *in vitro* models to screen potential drug candidates.

Neuron and astrocyte based cell models have been used to study neurodegenerative disease and CNS injuries. Neurons are carriers of electrochemical signals to the striatum that facilitates movement and these dopaminergic neurons are supported by the lowest number of astrocytes for any brain region, and hence vulnerable^5^. In fact, astrocytes are critical in the modulation of the neurotoxic effects of many inhibitors that induce experimental Parkinsonism and can invoke a neurotoxic to neurotrophic response. Indeed, astrocytes harbour an effective neuroprotective arsenal that includes neurotrophic factors and anti-oxidative stress molecules^6,7^. An intimate relationship exists between neurons and glia following response to injury. For example, during conditions of oxidative stress, neurons can utilise secreted astrocyte derived antioxidant molecules to reduce internal oxidative stress^8,9^.

Electrospun nanofibres scaffolds for 3D tissue engineering emerged during the 1990s^10,11^. 3D *in vitro* tissue models hold considerable value for a breadth of studies, from a basic understanding of neuronal-glial development through to the design of improved screening platforms for potential neuroprotective agents. Traditionally, *in vitro* neuronal cell culture has been performed using two-dimensional (2D) monolayer cultures on cell adherent tissue culture plastic (TCP) and have been criticised for not providing a native cellular environment, resulting in remodelling of cellular architecture and changes in gene expression^12,13^. The advantages of using 3D nanofibre scaffolds to mimic the *in vivo* environment are: (1) enhanced cellular architecture and physiology^14^, (2) greater cell to cell contact and interaction, with increased intercellular signalling^15^, (3) enhanced cell differentiation for complex tissue development^15^, (4) greater surface area and porosity with enhanced cell adhesion and improved access to metabolites and nutrients^16^. Cell behaviour is influenced by surface physicochemical properties including nanotopography, surface charge and protein adsorption/immobilisation^17^ and therefore nanofibres can be manipulated by copolymerization or by polymer blending of various synthetic and/or natural, non-biodegradable/biodegradable materials^18,19^.

In this study, novel electrospun 3D nanofibre scaffolds have been developed to improve discovery of neuroprotective agents for PD. The approach used electrospun PAN, a pure carbon based polymer and Jeffamine^®^ infused PAN. Jeffamine is a highly versatile polymer containing primary amino groups attached to the end of a polyether backbone generally based on propylene oxide (PO), ethylene oxide (EO) or a mixture of both (Huntsman, UK). Jeffamine polymer is commonly used as a copolymer to alter chemical and physical properties of other polymers.

SH-SY5Y human neuroblastoma and U-87MG human glioblastoma cell lines have been used to investigate several disorders including Parkinson’s disease, neurogenesis and other brain cell characteristics. Several studies have shown SH-SY5Y cells are capable of differentiating into mature dopaminergic neurons^20,21^ whereas U-87MG cells can be induced to differentiate into astrocytes^22^. Here, we have demonstrated that the chosen scaffolds are capable of harbouring these cell lines and support long-term cell survival, proliferation and differentiation using multiple differentiating agents. Cellularised nanoscaffolds were exposed to inhibitor treatments mimicking PD pathophysiology. Results confirmed that PAN nanoscaffolds prolonged the survival of SH-SY5Y cell cultures and PJ for U-87MG cultures, and that the 3D cultures have better proliferation and survival than 2D cultures.

## Results

### Rheometer measurement

**Figure 1:**
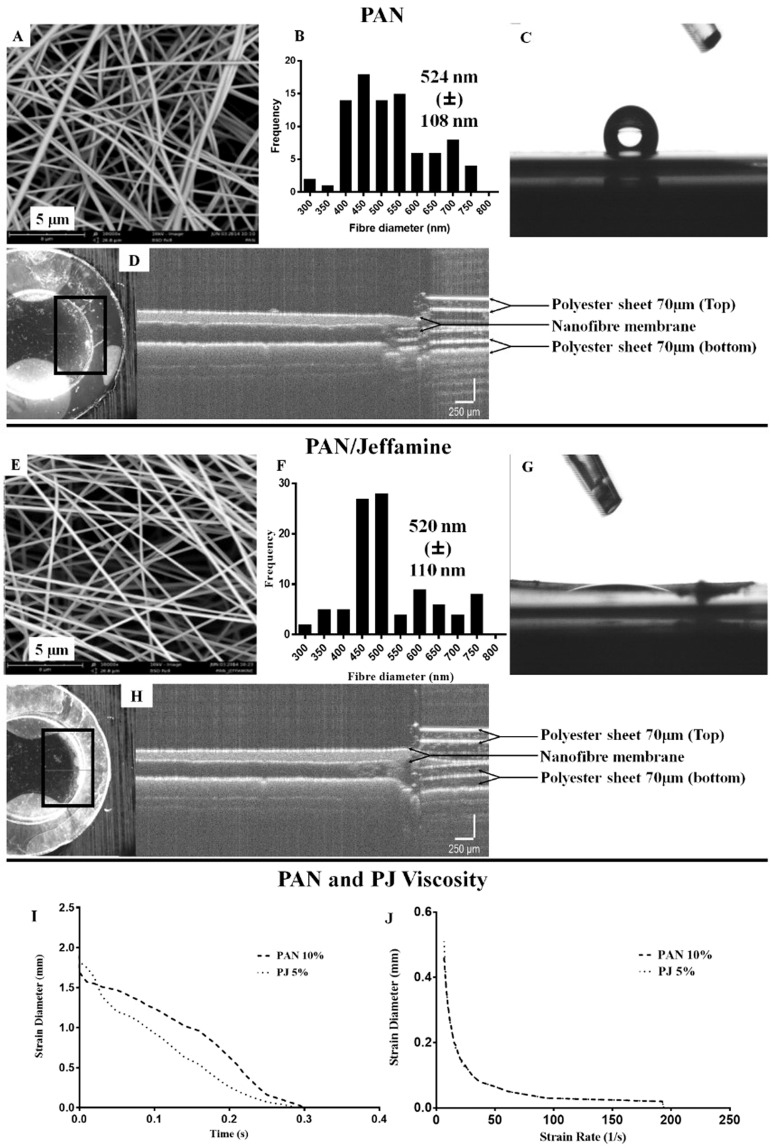
Characterisation of PAN and PJ scaffolds. Panels A–D and E–H represent PAN and PJ nanofibre scaffolds respectively. (**A,E**) show SEM analysis. (**B,F**) shows fibre diameter data. (**C,G**) shows contact angle measurement. (**D,H**) shows OCT image analysis for thickness. Graph (**I**) shows strain diameter vs time of PAN and PJ electrospinning formulation. Graph (**J**) shows the strain diameter vs the strain rate. Considering the two variables. PAN and PJ, the  rheology data was analysed using unpaired t-test with Welch’s correction and show normal distributions with equal variance. The Welch’s t-test was used for unequal variances, but the assumption of normality was maintained.

The extensional viscosity of both PAN and PJ electrospinning formulations were measured using the HAAKE- CaBER™ Rheometer. The results are important as part of an in process monitoring check on the molecular weight distribution of the PAN polymer used to make the batch. Higher average molecular weight electrospinning formulations have higher viscosity than lower. Subsequently, higher viscosity formulations produce nanofibres with larger diameters than lower for the same electrospinning set up and process parameters as well as the potential for incomplete desolvation of the nanofibre during synthesis which results in solvent welds where one nanofibre touches another. Hence varying viscosities between batches affects the cellular micro-environment and cell response. For an *in vitro* model it is important to produce consistent 3D nanofibre scaffolds from one batch to another. For PAN the shear viscosity was 0.7102 Pa.s with a breakup time of 0.298 seconds (Fig. 1I) and shear viscosity of 0.7030 Pa.s with breakup time of 0.218 seconds for PJ (Fig. 1J).

### Electrospun PAN and PJ nanofibres

Using the Phenom SEM FiberMetric software, 100 different measurements were analysed for each sample. The data is represented in an even mean distribution among the one hundred measurements with standard error and standard error mean (±). The average fibre diameters for PAN and PJ were 524 ± 108 nm and 520 ± 110 nm, respectively (Fig. 1B,F). Measurements from optical coherence tomography (OCT) data determined the average thickness of the scaffolds to be 30 µm for PAN and 33.5 µm for PJ (Fig. 1D,H). Contact angle values were 112.7° for PAN and 36.1° for PJ. Hence the PAN nanoscaffolds are hydrophobic and PJ hydrophilic (Fig. 1C,G).

**Figure 2:**
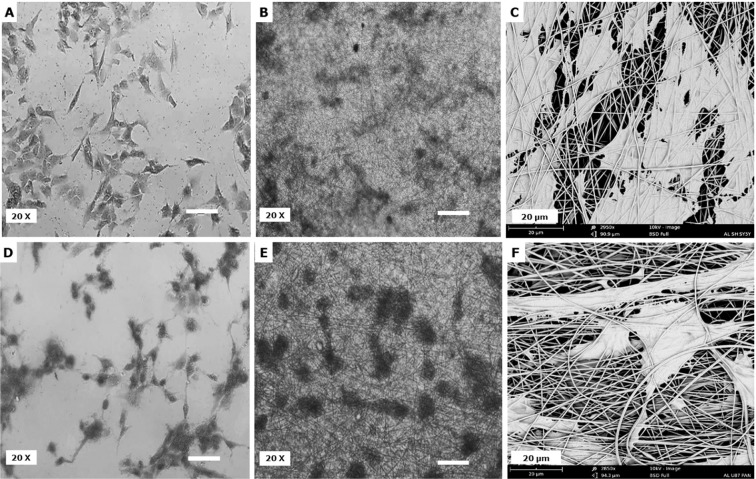
Neural cell attachment to scaffolds examined by Coomassie blue staining and SEM. Panels (**A–C**) show SH-SY5Y cells and (**D–F**) are U-87 MG cells. (**A,D**) Show light microscope image of Coomassie-stained cells on TCP and (**B,E**) on fibres. Panels (**C,F**) show SEM images of SH-SY5Y and U-87 MG cells on PAN and PJ, respectively. Cells were seeded at a density of 5 × 10^4^ cells/well for both TCP and fibre. (**A,B,D,E**) Scale bar represents 100 µm.

### Culture of neural cells on nanoscaffolds

Coomassie stained cultures visualised using a light microscope (Nikon Eclipse TS100) showed that neural cells could be cultured successfully on PAN and PJ fibres. Morphology of cells was also observed using SEM. Results confirmed that the cells were able to attach to both types of polymer (Fig. 2A,B,D and E). Indeed, SEM images showed both cell types were able to grow within the fibres, adapting to the 3D structure (Fig. 2C,F).

### Growth and proliferation of neural cells on nanofibres

**Figure 3:**
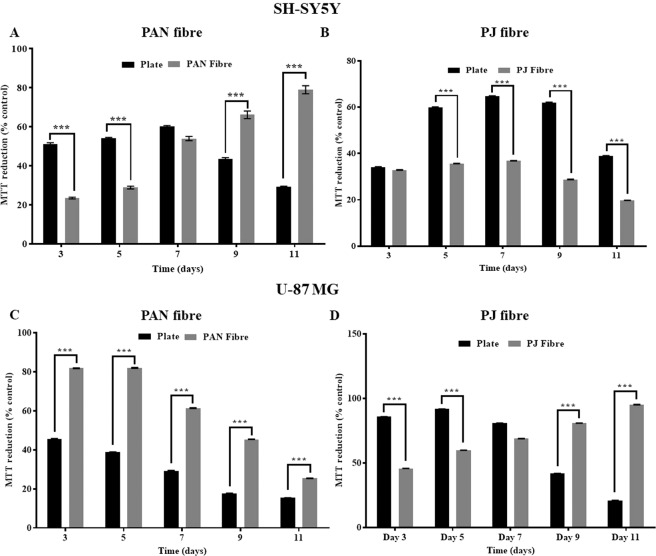
Analysis of MTT reduction by neural cell lines grown on fibres and TCP. MTT reduction assays were performed to compare cell growth and metabolic activity on fibres and TCP for 11 days. Shown are SH-SY5Y (**A,B**) and U-87 MG cells (**C,D**) cultured on PAN (**A,C**) and PJ fibres (**B,D**). SH-SY5Y and U-87MG cells grown on PAN and PJ fibres, respectively showed a gradual increase in MTT reduction, whereas SH-SY5Y on PJ and U-87MG on PAN showed a gradual fall in metabolic activity. Cells were seeded at a density of 5 × 10^4^ cells/well for both TCP and fibre. Values were converted to percentage of control (day 1) and are expressed as the % MTT reduction ± SEM where n = 6*, Statistical comparison was performed for plate vs fibres using the Two-Way ANOVA with Sidak’s multiple comparisons tests where *p < 0.05, **p < 0.01, ***p < 0.001.

The results show that SH-SY5Y cells were able to proliferate and survive longer on PAN fibres (Fig. 3A) compared to PJ fibres (Fig. 3B), while the rate of proliferation was reduced on fibres in comparison to culture plastic. In contrast, U-87 MG cells were able to proliferate and survive longer on PJ (Fig. 3D) (Fig fibres compared to PAN fibres (Fig. 3C). Similarly, the rate of proliferation was reduced on the PJ fibres compared to tissue culture plastic (TCP). It was noticed that cells grown on TCP entered log phase from day 3 and remained in the stationary phase for day 5 and 7, and subsequently began to die from day 9. Similar profiles were observed for SH-SY5Y cells cultured on PJ andU-87MG on PAN. SH-SY5Y on PAN and U-87MG on PJ (Fig. 3A,D) showed a steady increase and continued to proliferate within the log phase for 11 days.

### Differentiation of SH-SY5Y and U-87MG

**Figure 4:**
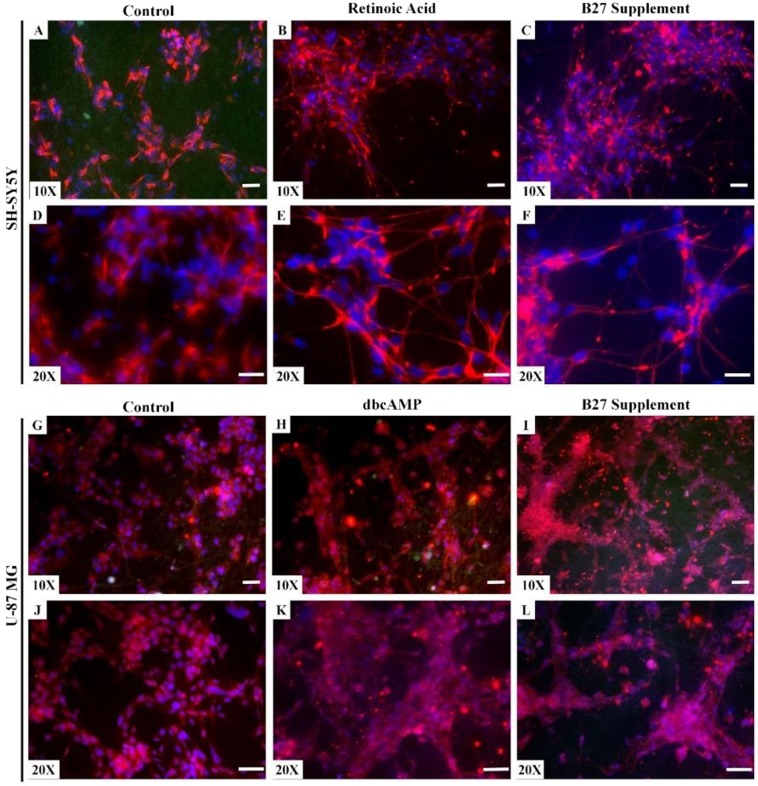
Immunofluorescence analysis of neural cell differentiation. SH-SY5Y (**A–F**) and U-87MG (**G–L**) cells were induced to differentiate on both PAN and PJ fibres respectively. SH-SY5Y cells were induced to differentiate with RA and B27 supplement. U-87MG were induced to differentiate with dbcAMP and B27 supplement. Red represents βIII tubulin (Panels A–F) and GFAP (Panels G–L), Blue represents DAPI nuclei stain, and green represents autofluoresence from nanofibres. Cells were seeded at a density of 5 × 10^4^ cells/well for both TCP and fibre. Scale bar represents 100 µm.

Cells were induced to differentiate using three different agents known to induce neural differentiation. Immunofluorescence images obtained from differentiating cells showed that neuronal SH-SY5Y cells were able to extend neurites on PAN fibres and that these cellular processes were stained positive for βIII tubulin, a mature neuronal marker. Both retinoic acid (RA) (Fig. 4B,E) and B27 (Fig. 4C,F) supplement showed similar results with extended βIII tubulin positive neurites compared to the non-treated control (Fig. 4A,D). However, on PJ fibres SH-SY5Y cells struggled to grow and differentiate. In contrast, U-87MG cells cultured on PJ showed improved cell growth and differentiation as highlighted by GFAP positive staining of cel-lular extensions showing induced astrocyte differentiation. U-87MG cells struggled to grow and differentiate in PAN fibres. GFAP was observed in U-87MG exposed to both dbcAMP (Fig. 4H,K) and B27 supplement (Fig. 4I,L) compared to the undifferentiated control (Fig. 4G,J).

### Expression levels of differentiation markers

**Figure 5:**
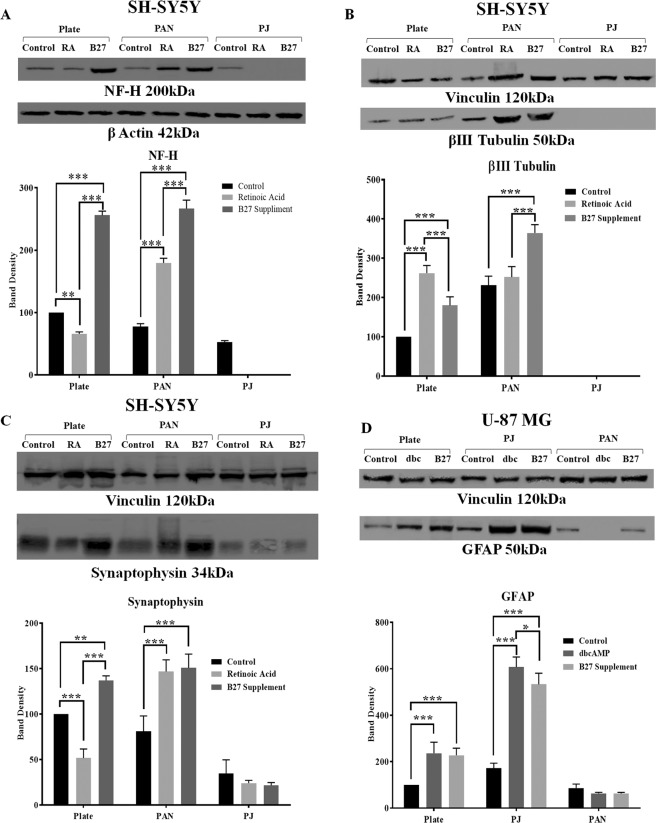
Expression of differentiation protein markers on Western blots. Marker protein levels were determined in lysates of SH-SY5Y (**A–C**) and U-87MG (**D**) cells cultured on both PAN and PJ fibres determined by western blot and densitometry analysis. β-actin at 42 kDa was used as loading control for NF-H and vinculin at 120 kDa for βIII tubulin, synaptophysin and GFAP. Densitometry values obtained were normalised against loading control. Results are expressed at the mean percentage ± SEM (n = 3). Asterisk (*) indicates changes on fibres that are significantly different (*p < 0.05, **p < 0.01 or ***p < 0.001) compared to TCP for undifferentiated (control) and the differentating agents were determined by Two-Way ANOVA with Sidak’s multiple comparisons tests.

Levels of multiple differentiation markers were examined using western blotting. Phosphorylated neurofilament heavy chain (axon and neuronal maturity marker), βIII tubulin (mature neuronal marker) and synaptophysin (synaptic and neuronal network maturity marker) were measured for SH-SY5Y cells and GFAP expression for U-87MG cells. To identify the markers of differentiation, both cell types were induced to differentiate on nanofibres and plates for seven days. Cell lysates were prepared, and the expression levels of the different proteins were compared. Our results indicate that expression of phosphorylated neurofilament-H (200 kDa), βIII tubulin (50 kDa) and synaptophysin (34 kDa) increased when cells were differentiated on PAN fibre compared to cells cultured on TCP (Fig. 5A–C). Poor expression of these proteins was observed from cells differentiated on PJ fibres. U-87MG cell extracts exhibited high expression of GFAP (50 kDa) on PJ fibre compared to TCP while low expression of GFAP was observed in undifferentiated (control) and B27 supplementation on PJ fibre (Fig. 5D). Both cell types expressed higher levels of neural marker protein when differentiated with B27 supplementation compared to RA and dbcAMP.

### Effects of PD mimetics on undifferentiated SH-SY5Y and U-87 MG cells

**Figure 6:**
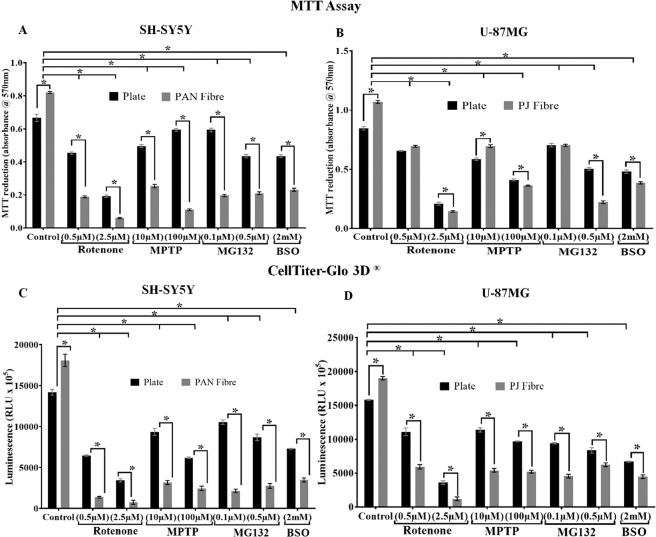
Effect of PD mimetics on mitotic SH-SY5Y and U-87MG cells. SH-SY5Y (**A,C**) and U-87MG (**B,D**) cells were cultured on PAN and PJ fibres and exposed to inhibitors for 72 hours as indicated. Cells were seeded at a density of 5 × 10^4^ cells/well for both TCP and fibre. The effect of inhibitors was compared between plate vs fibres and control vs treatment, using MTT reduction (**A,B**) and CellTiter-Glo^®^ 3D (**C,D**) assays. Results are expressed at the mean absorbance ± SEM for MTT (n = 6) and mean luminescence ± SEM (n = 3) for CellTiter-Glo 3D^®^. *P < 0.05 against plate vs fibres and control vs treatment for plate and fibres was determined by Two-Way ANOVA with Sidak’s multiple comparisons tests.

Different treatments capable of mimicking PD paradigms (namely mitochondrial impairment, glutathione depletion and proteasome inhibition) were incubated with SH-SY5Y and U-87MG cultured on PAN and PJ, respectively for 72 hours. The effect of the treatments on cells cultured on both nanofibres and plate were examined by analysing cell viability using both MTT reduction and CellTiter-Glo 3D^®^ assays (Fig. 6). The PD mimetic treatments used in this study were rotenone and MPTP to induce complex 1 inhibition, MG132 inducing proteasome inhibition and BSO, a glutamylcysteine synthetase inhibitor, to reduce glutathione levels. The results indicated that the cells exposed to treatments on nanofibres were more sensitive to the toxins compared to those grown on TCP, as they showed increased susceptibility to cell death.

### Effects of PD mimetics in a co-culture model with differentiated cells

**Figure 7:**
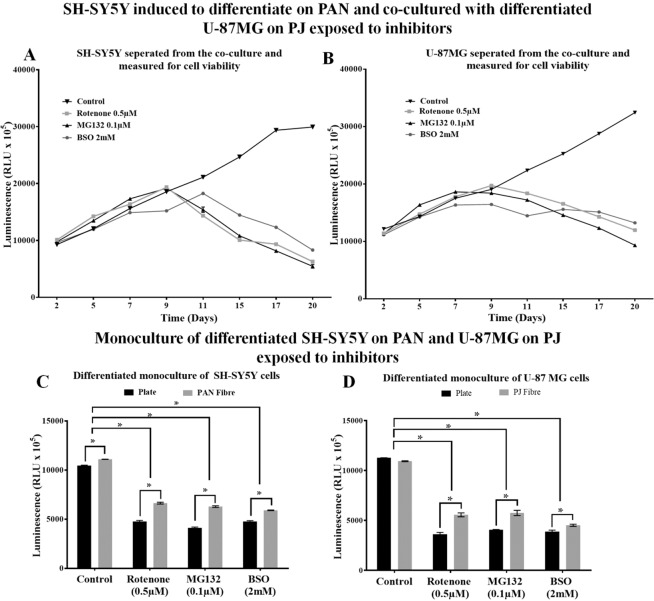
Effect of inhibitors on SH-SY5Y and U-87MG cells in a co-culture model. Neuronal and astrocyte viability was measured after 5 days differentiation in co-culture followed by exposure to inhibitors. Differentiated SH-SY5Y (**A**) and U-87MG (**B**) separated from the co-culture every 72 hours and measured for 20 days. Neuronal (**C**) and astrocyte (**D**) cultured under same conditions as individual cultures for 72 hours. Cells were seeded at a density of 5 × 10^4^ cells/well for both TCP and fibre. In Figure A no significant difference in the data was observed for Control vs. MG132 0.1 µM on day2, and Control vs. BSO 2 mM on day 2 and 5. In Figure B no significant difference in the data was observed for Control vs Rotenone (0.5 µM) on day 2 and Control vs BSO 2 mM on day 5. All other control vs treatments express a significant difference with a P value < 0.05. Results are expressed in mean luminescence value ± SEM (n = 3). Significant differences between control vs treatment for each measurement were determined by Two-Way ANOVA with Sidak’s multiple comparisons tests (*P < 0.05).

To investigate the ability of glial cells to support neuronal cells, toxicity tests were performed using a co-culture model of mature neurons from SH-SY5Ycells and astrocytes differentiated from U-87 MG cells (as explained in Section 4.10). Both cell types were differentiated using the B27 supplement and exposed to low levels of PD mimicking inhibitors. Our results showed that neurons on PAN fibres have prolonged survival in the presence of inhibitors when cultured as a co-culture along with astrocytes (Fig. 7A). Similar effects were also observed in astrocytes cultured on PJ fibres (Fig. 7B). Both cell types showed prolonged steady survival for nine days and very gradual cell death after day 11. When monocultures of differentiated SH SY5Y and U-87MG on nanofibre scaffolds were exposed to inhibitors for 72 hours, both cell types showed a heightened sensitivity to the different inhibitors but also exhibited a less cell death compared to TCP (Fig. 7C,D). In Fig. 7A,B show that both cells grown in co-culture together can grow for a period of 20 days (control). However, in the presence of inhibitors viability was compromised from day 11 for both cell types. In comparison, when cells were grown in a single culture (Fig. 7C,D) cell viability was compromised from early as day 3. Further exposure resulted in complete loss of viability, and therefore data beyond day 3 for this single culture experiment is not shown.

## Discussion

Nanofibre scaffolds have the ability to support and provide a suitable habitat for cells to attach, proliferate and differentiate^23^. Non-degradable, synthetic PAN nanofibres scaffolds were used in this study because their mechanical properties enable robust cell cultures to be produced. Jeffamine® ED-2003, a polyetheramine was mixed with PAN and N,N-dimethylformamide (DMF), then electrospun to produce PJ nanofibres. Rheometer measurements clearly showed that both PAN and PJ formulations have similar extensional viscosity and very similar strain breakup time. (Fig. 1I,J). Conditions were optimised in respect of polymer preparation, temperature, humidity, flow rate and the voltage to produce fibres with mean diameters between 500–600 nm, pore size of ~4000 nm^2^ and the scaffolds thicknesses of 30 µm and 33.5 µm for PAN and PJ, respectively (Fig. 1). The contact angle measurement using water drop test confirmed PAN scaffolds are hydrophobic^24^ and addition of 5% Jeffamine® changed the physical property of the fibres to hydrophilic (Fig. 1C,G). All scaffolds were sterilised using a 30-minute wash in 70% ethanol, with < 1% increase in the fibre diameter observed for both and this method showed no other major changes in the physical or chemical structure of the nanofibres. (Shown in supplementary data Figs. 1 and 2).

SH-SY5Y human neuroblastoma and U-87MG human glioblastoma cells were used to investigate the ability of the synthetic nanofibre scaffolds to facilitate cell adhesion, proliferation & differentiation. The results show that PAN and PJ nanofibres are suitable biomaterials to produce random nanofibre scaffolds that support neuronal and glial cells, respectively. The biomaterial preference for each cell type was evident in each cell type’s ability to attach and grow over a period of 11 days on nanofibre scaffolds. In both cases, cell growth and survival were maintained for longer on nanofibre scaffolds compared to conventional TCP. Several studies have reported the benefits and enhancement of cell metabolism when cultured on the 3D nanofibrillar surface^25,26^. The preferential attachment and growth of glial cells to PJ rather than PAN highlight potential differences in the ECM, which promotes the adhesion and proliferation of cells^27,28^. The composition of nanomaterials influences cell growth & survival due to topographic and biochemical cues induced by their unique physical and chemical properties^29^. When growing co-cultures, it is potentially more desirable to culture cells onto 3D scaffolds that are capable of mimicking the native ECM. The improved growth and survival of both cells types could also be attributed to the physical properties of the nanofibres and fully suspended, highly porous, 3D nanofibres structures allows cells greater access to nutrients as well as toxins compared to cells cultured on 2D TCP^30,31^.

Comparative information about neuronal and glial cells cultured on PAN and PJ fibres was not previously available in the literature. Our data confirm that both glial and neuronal cells are capable of attaching to and proliferating on PAN and PJ fibres, showing the ability to differentiate on specific nanofibres. SH-SY5Y cells had a preference for PAN fibres whereas U-87MG cells had a preference for PJ. It may be that hydrophobic surfaces in PAN scaffolds facilitate cell:cell interactions in neuronal cultures in a manner that favours neuronal differentiation, while a more hydrophilic scaffold favours glial cell attachment and differentiation. Further work will help to establish the molecular basis of these phenomena. Prolonged survival and proliferation of neuronal and glial cells on both PAN and PJ was investigated by allowing the cells to grow on these materials for 11 days. The cell viability data (Fig. 3) obtained from both fibres types and plate showed that the cells were able to survive longer on fibres than on TCP. This may be due to the increased cell membrane surface area available to access nutrients and gases from the medium. SH-SY5Y cells showed prolonged survival on PAN and U-87MG on PJ fibres, respectively. The ability of these cells to adapt to specific surfaces opens up a range of possibilities such as targeted cell growth and cell channelling.

SH-SY5Y cells were induced to differentiate into matured neurons showing extended axonal growth and expressing high levels of the neuronal marker βIII tubulin, whereas U-87 MG cell were induced to differentiate to astrocytes expressing high levels of the astrocyte-specific intermediate filament protein GFAP. Both cell types were exposed to multiple differentiation-inducing agents including RA for SH-SY5Y and dbcAMP for U-87MG cells^32–34^. The B27 supplement is a commercially available product that is commonly used for the survival of primary neurons and neural cell differentiation^35,36^ and was used in this study as an alternative differentiating agent. Several components in the B27 supplement have shown the ability to support healthy neuron growth including neurite development, synapse formation and neurotransmitter release in primary cells^37^. We examined whether B27 could promote axonal generation in SH-SY5Y cells and to differentiate U-87MG cells into astrocytes expressing high levels of GFAP. Our results showed that both cell lines were capable of differentiating on their preferred fibre scaffolds. As determined by indirect immunofluorescence, SH-SY5Y cells showed high expression of the neuronal markers βIII tubulin, phosphorylated neurofilament heavy chain on PAN fibres and U-87MG cells expressed higher levels of the astrocyte specific intermediate filament protein GFAP and exhibited star-shaped astrocytic morphology on PJ (Fig. 4). The increased levels of expression of these proteins and the neuronal marker synaptophysin were also confirmed in their respective cell types using Western blot analysis^38–40^. All protein biomarkers showed increased levels when cells were induced to differentiate on their preferred nanofibre scaffold. Furthermore, these levels were elevated in response to either dbcAMP or B27 supplementation (Fig. 5D).

While neuronal degeneration is a major event in PD, astrocytes are known to play a major role both in neuroprotection and the inflammatory response in PD^41–43^. Therefore, after identifying the ability of both cell types to survive and differentiate with respect to fibre type, it was important to determine the response of both SH-SY5Y and U-87MG cells when exposed to PD mimetics compared to cells grown on TCP (Fig. 6). Complex1 impairment, proteasome inhibition, and glutamylcysteine synthetase inhibition leading to neuronal degeneration are common pathophysiological responses observed and studied in PD^44–47^. Rotenone and MPTP are commonly used to induce complex 1 inhibition^48,49^, MG 132 mimics proteasome inhibition^50^ and BSO promotes glutamylcysteine synthetase inhibition^47^. We used rotenone, MPTP, MG132 and BSO to create PD-like conditions in SH-SY5Y and U-87MG cells cultured on both TCP and PAN/PJ fibres. Our results showed that the cells on fibres exhibited higher sensitivities to the different PD mimetics compared to cells grown on conventional TCP. This may be due to the increased surface area of cell membranes which is able to interact with the inhibitors on fibres compared to cells cultured on a TCP.

Neuronal and glial cell ratios differ in human brain regions^51^. Several studies have also shown that astrocytes support neurons and influence synaptic transmission, plasticity and neuronal protection^52^. To understand the ability of astrocytes to support neuronal cell survival against PD mimetics we designed a co-culture model using two separate nanofibre scaffolds. SH-SY5Y cells were cultured in PAN fibres, and U-87MG cells were cultured on PJ fibres. Both cell lines were cultured for 48 hours and induced to differentiate using the B27 supplement for five days followed by exposure to low concentrations of inhibitors. The viability of both cell types on plate and fibres was examined individually using CellTiter-Glo^®^ 3D. The results indicated that neuronal cells exhibit improved survival rates when cultured with astrocytes (Fig. 7A,B). Neuronal cells showed prolonged survival against the inhibitors for nine days with a gradual loss of viability only seen from the 11^th^ day. This was repeated in astrocyte cell populations. The chronic co-culture model has shown how two different types of cells can be co-cultured on different surfaces which can be used to recreate a suitable chronic cell culture model of PD. When co-cultured on nanofibres, neuronal cells had a lower rate of cell death in response to inhibitors. Studies have shown astrocytes can provide the neurotrophic factor like glial cell line-derived neurotrophic factor (GDNF) or brain-derived neurotrophic factor (BDNF)^53,54^ and the nanofibres may provide a robust structural support that helps to prolong cell survival. Several studies have also shown that glial cell line-derived neurotrophic factors can support neuronal cell line growth and are very useful to study neurodegenerative diseases like PD^55,56^. When differentiating neuronal and glial cells were exposed to PD mimetics individually, they exhibited the same response as the undifferentiated cell toxicity test as shown in Section 2.7 (Fig. 7C,D). Both differentiated neurons and astrocytes were sensitive to the inhibitors, but the rate of cell death was lower compared to TCP (Fig. 7A,B). It was interesting to note that, in contrast to the effects observed on mitotic monocultures, neuronal and glial cells differentiated separately on fibres were less sensitive to PD mimetics than cells grown on TCP. Further work will help to determine whether this reflects differences in the metabolic demand of neural progenitors compared to differentiated cells.

The nanofibre based co-culture model described in the current work represents a long-term 3D differentiated culture model that responds to PD mimetics that have the potential to be used to investigate potential neuroprotective agents from both a neuronal and glial perspective. A major benefit of this model compared to 2D co-culture models of PD, is that neuronal and glial cell types can be co-cultured during exposure then be much more easily separated for analysis at a molecular or cellular level to detect cell type- specific changes. The enhanced neuroprotective effect against inhibitor treatment observed in scaffold co-cultures, compared to both 3D and 2D monocultures, also suggests that our 3D model enhances the natural ability of neural cells to afford protection to neighbouring cells, providing a strong foundation for longer term chronic exposure studies *in vitro*.

Using 3D nanofibres scaffolds populated with differentiated neural cells to investigate PD could lead to a breakthrough in understanding the importance of simulating the natural habitat of cells, using nanofibres to support the survival of different cell types. Taken further, multiple treatments could be investigated and scaled up manufacture of suspended nanofibres scaffolds in well plates would improve high content screening models used in drug discovery and to identify possible biomarkers linked to the loss of neuronal cells in Parkinson’s disease.

## Methods

### Electrospinning

PAN, M_w_ 150,000 (Sigma Aldrich, UK) was dissolved in 20 ml N,N-dimethylformamide(DMF) (Fisher Scientific, UK) to form 10 wt% PAN/DMF solution and 5 wt% Jeffamine® ED-2003 (Huntsman, Holland BV) in 10 wt% PAN/DMF for PJ solution and heated overnight, 50 °C, with constant stirring. Extensional viscosity was measured using HAAKE-CaBER™ 1 Capillary Breakup Extensional Rheometer (Thermo Scientific, UK). A 10 ml syringe was filled and connected to a 21 gauge blunt-end needle using 1/32” bore Polytetrafluoroethylene (PTFE) tubing. Polyester sheets 600 × 300 × 0.075 mm were laser cut to create 23 by 11 array of 10 mm diameter apertures. One sheet was wrapped around an electrically earthed collector drum. The needle to collector distance was set to 300 mm. A controlled environment of 20 °C and 65%RH was maintained throughout the spinning process. A syringe pump (New Era pump system, NY, USA) supplied 2.5 ml of solution at 1 ml/h to the electrospinning needle which was set at 20 kV for PAN and 16 kV for PJ, to form random fibres membranes. These were secured by aligning a second laser cut sheet to the coated sheet and joined using ultrasonic spot welding. Laser cutting segmented the array to form individual suspended nanofibres inserts compatible with 12 well plates.

### Characterisation of nanofibres

Both the integrity and morphology of electrospun nanofibres were analysed by sputter coating a gold conductive thin film using an Edwards S150B system, then imaging using a Phenom ProX scanning electron microscope (SEM). Diameter and pore size distributions were analysed using FiberMetric software. Overlapping fibres and out of focus fibres were manually deselected from the measured data. The thickness of the nanoscaffolds were measured using OCT and ImageJ. Contact angles were measured from high-speed video frames of 10µl-deionised water dropped on the centre of the scaffolds and the interaction of the water droplet recorded for 10 seconds. A water contact angle less than 90 degrees is a hydrophilic surface, greater than 90 degrees, a hydrophobic surface^57^.

### Cell culture

Nanofibre inserts were sterilised by soaking in 70% (v/v) ethanol/dH_2_O for 30 minutes, washed twice with sterile phosphate buffered saline (PBS), and equilibrated with cell culture medium before transferring to well plates. Human SH-SY5Y neuroblastoma and human U-87MG glioblastoma cells (American Type Culture Collection, Manassas, VA) were grown at 37 °C in 5%(v/v) CO_2_. SH-SY5Y cells were maintained in Dulbecco’s modified Eagle’s medium (DMEM)- F12 (Lonza, UK) medium supplemented with 10%(v/v) heat inactivated foetal bovine serum (FBS), 2mM L-glutamine (Lonza, UK), penicillin (100 units/ml), streptomycin (100 µg/ml) (Lonza, UK), 0.1 mM non-essential amino acids (Lonza, UK). U-87MG cells in Eagle’s minimal essential medium (EMEM) with 1 mM sodium pyruvate (Lonza, UK), and supplemented as for DMEM. On reaching 80% confluency, cells were trypsinised and seeded onto fibre membranes at a density of 5 × 10^4^ cells per insert in a 12 well plate.

### Cell attachment and proliferation assays

To evaluate the level of attachment and proliferation, cells were cultured for 48 hours on both PAN and PJ fibres and then fixed and stained with Coomassie Brilliant Blue dye (Sigma-Aldrich, UK) to visualise cell attachment. Both SH-SY5Y and U-87MG were cultured on PAN, PJ and TCP seeded at a density of 5 × 10^4^ cell/well. Proliferation and survival of cells on each fibre type and on TCP were measured for 11 days with medium changes every 48 hours. Each sample was tested for metabolic activity (indicative of proliferation and/or viability) using the 3-(4,5-dimethylthiazol-2-yl)-2,5-diphenyltetrazolium bromide (MTT) (Sigma Aldrich, UK) reduction assay^58,59^ every 48 hour period for 11 days. The metabolic activity measurement obtained was analysed by comparing it with the data measured 24 hours after cell seeding (day 1 control|). Cell growth on PAN and PJ fibres were also compared to that of cells cultured on TCP.

### Cell differentiation

Experiments were performed on both fibre types in 12 well plates and on poly-L-lysine coated coverslips (Bio Coat Inc., USA). SH-SY5Y and U-87MG cells were induced to differentiate by culturing at 5 × 10^4^ cells/well under normal conditions. Following recovery, both cell types were grown in serum-free medium. For SH-SY5Y cells, medium was supplemented with retinoic acid (RA; 1 µM) and B27 supplement (1% (v/v), whereas U-87MG cells were grown in serum-free medium supplemented with dibutyryl cyclic adenosine monophosphate (dbcAMP; 0.3 mM) and B27 Supplement (1% v/v). Cells were cultured at 37 °C in 5% (v/v) CO_2_ for five days. During differentiation, medium was changed every 48 hours.

### Cell imaging

#### Scanning electron microscopy

After the completion of experimental incubations, cells were fixed in 90% (v/v) methanol in Tris buffered saline (TBS: 50 mM Tris-Cl, 150 mM NaCl, pH 7.6) at −20 °C for 30 minutes. After fixation, cells were dehydrated by sequential incubation in ethanol (v/v) at 30%, 50%, 70%, 80%, 90% and 100% for 10 min with gentle agitation^60,61^. After the final dehydration step, samples were air-dried for 15 minutes prior to mounting on SEM stubs and sputter coated with a 10 nm thick gold layer. Samples were imaged with an SEM at 10 kV.

#### Immunofluorescence

Following 5 days of cell differentiation, the medium was removed and cells were fixed in 90% (v/v) methanol in TBS at −20 °C for 30 minutes^62^. SH-SY5Y and U-87MG cells were stained for βIII tubulin and glial fibrillary acidic protein (GFAP), respectively. Cells were permeabilised with 1% (v/v) Triton X-100 in TBS. Primary mouse monoclonal anti-βIII tubulin (1:2000; Sigma-Aldrich) and rabbit polyclonal anti-GFAP (1:500; New England Biolabs, UK) antibodies were diluted in 3% (w/v) bovine serum albumin in TBS and incubated at 4 °C overnight. Because PAN and PJ nanofibres autofluoresce emitting green light, Alexa Fluor^™^ 568 goat anti-mouse Ig for anti-βIII tubulin and Alexa Fluor™ 568 chicken anti-rabbit Ig for GFAP (both from Cell Signalling, UK) were used at 1:200 dilutions. Cells were mounted on glass slides and counterstained with 4′,6-diamidino-2-phenylindole dihydrochloride (DAPI; Vector Laboratories Ltd., UK).

### Western blot analysis

Cells were cultured and differentiated on each fibre type, then washed twice with PBS, transferred into a tube and solubilised with RIPA buffer (Sigma-Aldrich) supplemented with 0.2% (v/v) protease inhibitor cocktail (Sigma Aldrich). Samples were vortex-mixed to break the fibres and cultures apart and create a protein extract. For comparison, cell pellets were also obtained by scraping cell monolayers on TCP in 12 well plates, followed by centrifuging at 300 g for 5 minutes. Pellets were resuspended in RIPA buffer with 0.2% (v/v) protease inhibitor cocktail. The protein concentration of each sample was determined using the Mini Lowry protein estimation assay. A total of 20 µg of cell lysate protein was separated by sodium dodecyl sulphate polyacrylamide gel electrophoresis (SDS-PAGE) using a 10% (w/v) polyacrylamide resolving gel. Proteins were transferred electrophoretically onto a nitrocellulose membrane filter and blocked. Lysates from SH-SY5Y cells were probed with mouse monoclonal anti-phosphorylated neurofilament heavy chain (NF-H) SMI 34 diluted 1:100 (Bio Legend, UK), mouse anti-βIII –tubulin [2G10] diluted 1:1000 (Abcam, UK), and mouse anti-synaptophysin diluted 1:10000 (Abcam, UK). U-87MG cell lysates were probed with rabbit anti-GFAP diluted 1:500 (GeneTex, UK). Samples were also probed with mouse anti-β actin 1:1000 GT5512 (GeneTex, UK) as a loading control for SMI 34 and rabbit anti-vinculin 1:5000 (Abcam, UK ab73412) as loading control for βIII tubulin, synaptophysin and GFAP. The membranes were probed with horseradish peroxidase-conjugated goat anti-mouse or goat anti-rabbit immunoglobulin, at 1:500. Antibody binding was detected by enhanced chemiluminescence and images were obtained using a Fujifilm LAS-4000 scanner) and band intensities quantified using Advanced Image Data Analyser Software (Raytek, Sheffield, UK)

### Cell treatments with different PD mimetics

Biochemical abnormalities found in PD can be induced with inhibitors that impair the functionality of the cells^63^. Mitotic SH-SY5Y and U-87MG cells were incubated at a density of 5 × 10^4^ cells/well on fibres and on plates. After 24 hours, the scaffolds were transferred to new plates. Both types were incubated for 24 hours at 37 °C with 5% (v/v) CO_2_ supply. After 48 hours, the medium was replaced with fresh medium containing PD mimetics at different concentrations. The inhibitors used were rotenone (0.5 µM and 2.5 µM) and 1-methyl-4-phenyl-1,2,3,4-tetrahydropyridine (MPTP) (10 µM and 100 µM) to induce complex 1 inhibition^64,65^, MG132 (0.1 µM and 0.5 µM) inducing proteasome inhibition^66^ and BSO (2.0 mM),a glutamylcysteine synthetase inhibitor, to reduce glutathione levels^67^. These were incubated under normal conditions for 72 hours and were tested for viability using the MTT and CellTiter-Glo® 3D assays (Promega UK).

### Co-culture model to test PD mimetics

**Figure 8:**
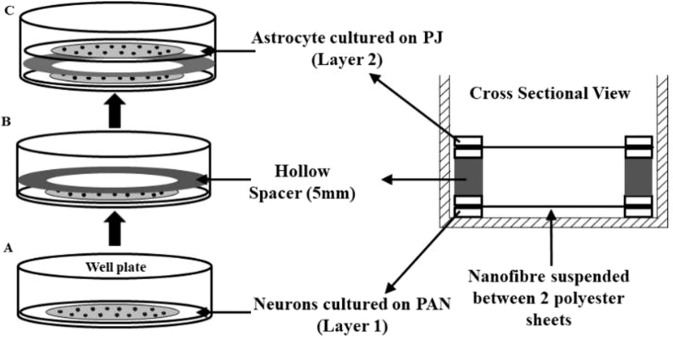
Schematic of co-culture model. (**A**) Neuronal cells cultured on PAN fibres. (**B**) Hollow acrylic spacer (**C**) Astrocytes on PJ fibres.

The effects of inhibitors on SH-SY5Y cells co-cultured in the presence of U-87 MG was investigated. SH-SY5Y cells were cultured on PAN and U-87MG on PJ scaffolds. Initially, both cell lines were seeded individually at a density of 5 × 10^4^ cells/ml, cultured for 48 hours and induced to differentiate with B27 supplement for five days. After five days, fibres scaffolds were arranged as shown in Fig. 8 and 0.5 µM rotenone, 0.1 µM MG132 and 2 mM BSO were added to the co-culture, which was exposed for 20 days to recreate the longer term, chronic model. Every 72 hours the fibres were transferred from each well into a fresh culture dish to measure viability and new medium with inhibitors was replaced for the remaining wells. Cell viability was examined for each fibre type using CellTiter-Glo® 3D. Simultaneously, under the same condition monoculture of both differentiated SH-SY5Y and U-87MG on PAN and PJ respectively were exposed to the same concentrations of inhibitors and cell viability was determined.

### Statistical analysis

Unless otherwise specified in figure legends, values obtained for proliferation and viability assessments were converted to a percentage of the corresponding control. The data obtained were normally distributed (as ascertained by the Kolmogorov-Smirnov Test for Normality). Statistical analysis was performed using the Two-Way ANOVA with Sidak’s multiple comparisons tests. The two-way ANOVA compares the mean differences between groups that have been split into two independent variables (Plate and Fibre) and the Sidak multiple comparisons was applied to compare each time point against the two variables. All data presented as mean ± the standard deviation (SD). Statistical analysis was performed on all datasets, and all analysis was conducted using the Graph Pad Prism for Windows Version 7.02. P-values that were considered statistically significant and were indicated within figures as *p < 0.05, **p < 0.01 or ***p < 0.001.

## Supplementary information

Supplementary Information

## Data Availability

The raw/processed data required to reproduce these findings are available upon request.

## References

[1] Davie CA (2008). A review of Parkinson’s disease. Br. Med. Bull..

[2] Bernheimer H, Birkmayer W, Hornykiewicz O, Jellinger K, Seitelberger F (1973). 1. Brain dopamine and the syndromes of Parkinson and Huntington Clinical, morphological and neurochemical correlations. J. Neurol. Sci..

[3] Lindvall O, Kokaia Z (2009). Prospects of stem cell therapy for replacing dopamine neurons in Parkinson’s disease. Trends in Pharmacological Sciences.

[4] Kusena J, Wilson S, Thomas R, McCall M (2018). 188 - Translational requirements for enhanced characterisation of manufactured dopaminergic neurons for the treatment of Parkinson’s disease. Cytotherapy.

[5] Hammond, C., Cayre, M., Panatier, A. & Avignone, E. Neuron–glial cell cooperation. *Cellular and Molecular Neurophysiology*, **25** (2014).

[6] Pla A, Pascual M, Guerri C (2016). Autophagy constitutes a protective mechanism against ethanol toxicity in mouse astrocytes and neurons. PloS one.

[7] Hass DT, Barnstable CJ (2016). Uncoupling protein 2 in the glial response to stress: implications for neuroprotection. Neural Regeneration Research.

[8] Peuchen S (1997). Interrelationships between astrocyte function, oxidative stress and antioxidant status within the central nervous system. Prog. Neurobiol..

[9] Miyazaki I (2011). Astrocyte-derived metallothionein protects dopaminergic neurons from dopamine quinone toxicity. Glia.

[10] Doshi J, Reneker DH (1995). Electrospinning process and applications of electrospun fibres. J. Electrostatics.

[11] Reneker DH, Yarin AL (2008). Electrospinning jets and polymer nanofibres. Polymer.

[12] Vergani L, Grattarola M, Nicolini C (2004). Modifications of chromatin structure and gene expression following induced alterations of cellular shape. Int. J. Biochem. Cell. Biol..

[13] Sever M, Gunay G, Guler MO, Tekinay AB (2018). Tenascin-C derived signaling induces neuronal differentiation in a three-dimensional peptide nanofibre gel. Biomaterials science.

[14] Knight, E. & Przyborski, S. Advances in 3D cell culture technologies enabling tissue-like structures to be created *in vitro*. *J. Anat*. (2014). 10.1111/joa.12257 PMC4694114 25411113

[15] Vasita R, Katti DS (2006). Nanofibres and their applications in tissue engineering. Int. J. Nanomedicine.

[16] Wu J, Hong Y (2016). Enhancing cell infiltration of electrospun fibrous scaffolds in tissue regeneration. Bioactive Materials.

[17] Lee Y, Livingston Arinzeh T (2011). Electrospun nanofibrous materials for neural tissue engineering. Polymers.

[18] Cai Y (2011). Effects of nano-SiO_2_ on morphology, thermal energy storage, thermal stability, and combustion properties of electrospun lauric acid/PET ultrafine composite fibres as form-stable phase change materials. Appl. Energy.

[19] Mazinani S, Ajji A, Dubois C (2009). Morphology, structure and properties of conductive PS/CNT nanocomposite electrospun mat. Polymer.

[20] Avola R, Graziano ACE, Pannuzzo G, Albouchi F, Cardile V (2018). New insights on Parkinson’s disease from differentiation of SH-SY5Y into dopaminergic neurons: An involvement of aquaporin 4 and 9. Molecular and Cellular Neuroscience.

[21] Sang Q (2018). Curcumin Protects an SH-SY5Y Cell Model of Parkinson’s Disease Against Toxic Injury by Regulating HSP90. Cell. Physiol. Biochem..

[22] Xing F (2017). The anti-warburg effect elicited by the cAMP-PGC1α pathway drives differentiation of glioblastoma cells into astrocytes. Cell. reports.

[23] Chan B, Leong K (2008). Scaffolding in tissue engineering: general approaches and tissue-specific considerations. European spine journal.

[24] Feng L (2002). Super-hydrophobic surface of aligned polyacrylonitrile nanofibres. Angewandte Chemie International Edition.

[25] Farzaneh Z, Pournasr B, Ebrahimi M, Aghdami N, Baharvand H (2010). Enhanced functions of human embryonic stem cell-derived hepatocyte-like cells on three-dimensional nanofibrillar surfaces. Stem. Cell. Reviews and Reports.

[26] Mahairaki V (2010). Nanofibre matrices promote the neuronal differentiation of human embryonic stem cell-derived neural precursors *in vitro*. Tissue Engineering Part A.

[27] Frantz C, Stewart KM, Weaver VM (2010). The extracellular matrix at a glance. J. Cell. Sci..

[28] Hinz B (2015). The extracellular matrix and transforming growth factor-β1: Tale of a strained relationship. Matrix Biology.

[29] Chueng SD, Yang L, Zhang Y, Lee K (2016). Multidimensional nanomaterials for the control of stem cell fate. Nano Convergence.

[30] Duval K (2017). Modeling physiological events in 2D vs. 3D cell culture. Physiology.

[31] Joseph JS, Malindisa ST, Ntwasa M (2018). Two-Dimensional (2D) and Three-Dimensional (3D) Cell Culturing in Drug Discovery. Cell. Culture.

[32] Tiryaki VM, Ayres VM, Ahmed I, Shreiber DI (2015). Differentiation of reactive-like astrocytes cultured on nanofibrillar and comparative culture surfaces. Nanomedicine.

[33] Korecka JA (2013). Phenotypic characterization of retinoic acid differentiated SH-SY5Y cells by transcriptional profiling. PloS one.

[34] Fedoroff S, McAuley W, Houkle J, Devon R (1984). Astrocyte cell lineage. V. Similarity of astrocytes that form in the presence of dBcAMP in cultures to reactive astrocytes *in vivo*. J. Neurosci. Res..

[35] Roth S, Zhang S, Chiu J, Wirth EK, Schweizer U (2010). Development of a serum-free supplement for primary neuron culture reveals the interplay of selenium and vitamin E in neuronal survival. Journal of Trace Elements in Medicine and Biology.

[36] Kovalevich, J. & Langford, D. In *Neuronal Cell. Culture* 9–21 (Springer, 2013).

[37] Chen Y (2008). NS21: re-defined and modified supplement B27 for neuronal cultures. J. Neurosci. Methods.

[38] Gray E (2013). Accumulation of cortical hyperphosphorylated neurofilaments as a marker of neurodegeneration in multiple sclerosis. *Multiple Sclerosis*. Journal.

[39] Choi SH (2014). A three-dimensional human neural cell culture model of Alzheimer’s disease. Nature.

[40] Lee TT (2015). Inhibition of catechol-O-methyl transferase (COMT) by tolcapone restores reductions in microtubule-associated protein 2 (MAP2) and synaptophysin (SYP) following exposure of neuronal cells to neurotropic HIV. J. Neurovirol..

[41] O’Keeffe, G. W. & Sullivan, A. M. Evidence for dopaminergic axonal degeneration as an early pathological process in Parkinson’s disease. *Parkinsonism Relat. Disord*. (2018). 10.1016/j.parkreldis.2018.06.025 29934196

[42] Niranjan R (2014). The role of inflammatory and oxidative stress mechanisms in the pathogenesis of Parkinson’s disease: focus on astrocytes. Mol. Neurobiol..

[43] Miyazaki I, Asanuma M (2016). Serotonin 1A Receptors on Astrocytes as a Potential Target for the Treatment of Parkinson’s Disease. Curr. Med. Chem..

[44] Krebiehl G (2010). Reduced basal autophagy and impaired mitochondrial dynamics due to loss of Parkinson’s disease-associated protein DJ-1. PloS one.

[45] Mortiboys H, Johansen KK, Aasly JO, Bandmann O (2010). Mitochondrial impairment in patients with Parkinson disease with the G2019S mutation in LRRK2. Neurology.

[46] Xie W (2010). Proteasome inhibition modeling nigral neuron degeneration in Parkinson’s disease. J. Neurochem..

[47] Smeyne M, Smeyne RJ (2013). Glutathione metabolism and Parkinson’s disease. Free Radical Biology and Medicine.

[48] Innamorato NG (2010). Different susceptibility to the Parkinson’s toxin MPTP in mice lacking the redox master regulator Nrf2 or its target gene heme oxygenase-1. PLoS One.

[49] Tanner CM (2011). Rotenone, paraquat, and Parkinson’s disease. Environ. Health Perspect..

[50] Chan NC (2011). Broad activation of the ubiquitin-proteasome system by Parkin is critical for mitophagy. Hum. Mol. Genet..

[51] Azevedo FA (2009). Equal numbers of neuronal and nonneuronal cells make the human brain an isometrically scaled-up primate brain. J. Comp. Neurol..

[52] Hillen, A. E., Burbach, J. P. H. & Hol, E. M. Cell adhesion and matricellular support by astrocytes of the tripartite synapse. *Prog. Neurobiol*. (2018). 10.1016/j.pneurobio.2018.02.002 29444459

[53] Lindahl M, Saarma M, Lindholm P (2017). Unconventional neurotrophic factors CDNF and MANF: Structure, physiological functions and therapeutic potential. Neurobiol. Dis..

[54] Dougherty KD, Dreyfus CF, Black IB (2000). Brain-derived neurotrophic factor in astrocytes, oligodendrocytes, and microglia/macrophages after spinal cord injury. Neurobiol. Dis..

[55] Nishiguchi M (2003). Increase in secretion of glial cell line-derived neurotrophic factor from glial cell lines by inhibitors of vacuolar ATPase. Neurochem. Int..

[56] Shishkina TV (2018). Glial cell line-derived neurotrophic factor (GDNF) counteracts hypoxic damage to hippocampal neural network function *in vitro*. Brain Res..

[57] Förch, R., Schönherr, H., Schonherr, H. & Jenkins, A. T. A. In *Surface design: applications in bioscience and nanotechnology* (John Wiley & Sons, 2009).

[58] Mosmann T (1983). Rapid colorimetric assay for cellular growth and survival: Application to proliferation and cytotoxicity assays. Journal of Immunological Methods.

[59] Berridge MV, Herst PM, Tan AS (2005). Tetrazolium dyes as tools in cell biology: New insights into their cellular reduction. Biotechnology Annual Review.

[60] Braet F, De Zanger R, Wisse E (1997). Drying cells for SEM, AFM and TEM by hexamethyldisilazane: a study on hepatic endothelial cells. J. Microsc..

[61] Parameswaran S, Verma RS (2011). Scanning electron microscopy preparation protocol for differentiated stem cells. Anal. Biochem..

[62] Mey J, Schrage K, Wessels I, Vollpracht-Crijns I (2007). Effects of inflammatory cytokines IL-1β, IL-6, and TNFα on the intracellular localization of retinoid receptors in Schwann cells. Glia.

[63] Sherer TB (2002). An *in vitro* model of Parkinson’s disease: linking mitochondrial impairment to altered alpha-synuclein metabolism and oxidative damage. J. Neurosci..

[64] Gould, F. D., Gross, A., German, R. Z. & Richardson, J. R. Evidence of Oropharyngeal Dysfunction in Feeding in the Rat Rotenone Model of Parkinson’s Disease. *Parkinson’s Disease* **2018** (2018). 10.1155/2018/6537072 PMC5866867 29713446

[65] Qian F, Wang M, Wang J, Lu C (2018). Anthocyanin-Rich Blueberry Extract Ameliorates the Behavioral Deficits of MPTP-Induced Mouse Model of Parkinson’s Disease via Anti-Oxidative Mechanisms. *Yangtze*. Medicine.

[66] Zhang Q (2018). Cdk5 suppression blocks SIRT1 degradation via the ubiquitin-proteasome pathway in Parkinson’s disease models. Biochimica et Biophysica Acta (BBA)-General Subjects.

[67] Sian, J., Gerlach, M. & Riederer, P. In *Glutathione In The Nervous System* 287–304 (Routledge, 2018).

