# Supplementary material for: A co-culture nanofibre scaffold model of neural cell degeneration in relevance to Parkinson’s disease
Source: Sci Rep. 2020 Feb 17;10:2767. doi: 10.1038/s41598-020-59310-x (PMC7026118; doi:10.1038/s41598-020-59310-x)
Supplement: Supplementary file 1 — Supplementary Information [file 41598_2020_59310_MOESM1_ESM.docx]

A co-culture nanofibre scaffold model of neural cell degeneration in relevance to Parkinson’s disease

Joseph M. Chemmarappally* ^1,2^, Henry C N. Pegram^1,2^, Neranga Abeywickrama^1^, Enzo Fornari^1^, Alan J. Hargreaves^2^, Luigi A. De Girolamo^2^, Bob Stevens^† 1^

^1^ Innovations in Surfaces, Materials and Related Technologies (iSMART), College of Science and Technology, Nottingham Trent University, Clifton, UK, NG11 8NS

^2^ Interdisciplinary Biomedical Research Centre (IBRC), College of Science and Technology, Nottingham Trent University, Clifton, UK, NG11 8NS

**Supplementary data**

**
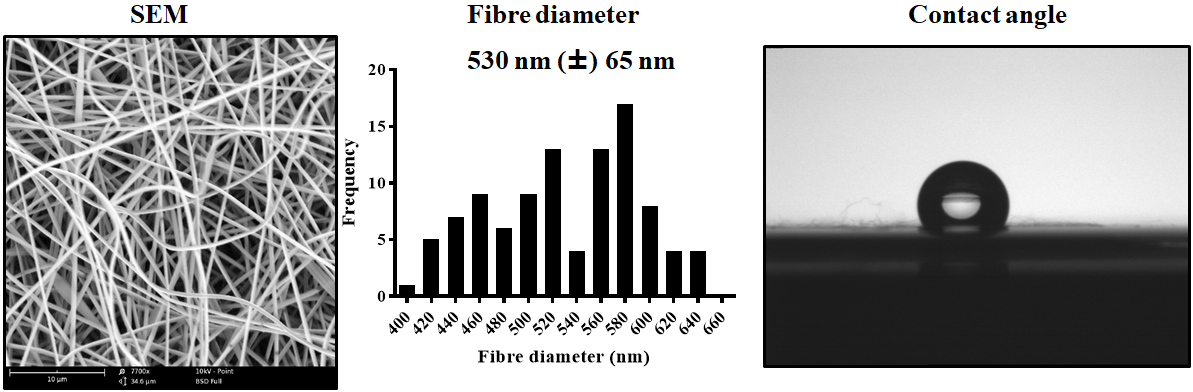
**

**Supplementary Figure 1: PAN property analysis after 30 minute ethanol sterilisation.** The scaffolds were soaked in ethanol for 30 minutes and air dried. Then scaffolds were analysed for any change in the physical and surface property using SEM analysis and contact angle measurement. No change in the property was observed after the ethanol exposure.

**
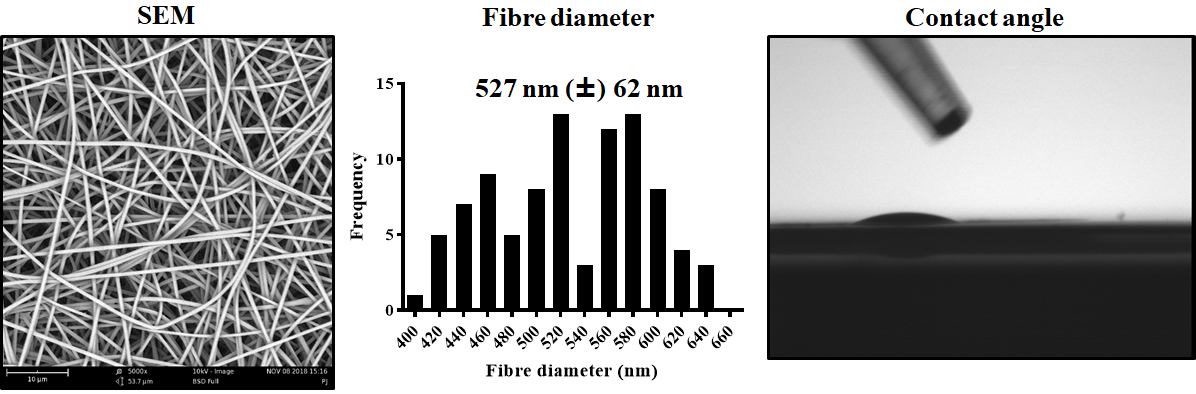
**

**Supplementary Figure 2: PJ property analysis after 30 minute ethanol sterilisation.** The scaffolds were soaked in ethanol for 30 minutes and air dried. Then scaffolds were analysed for any change in the physical and surface property using SEM analysis and contact angle measurement. No change in the property was observed after the ethanol exposure.
